# Editorial: Innovative approaches in pediatric surgical oncology

**DOI:** 10.3389/fped.2022.989822

**Published:** 2022-08-04

**Authors:** Luca Pio, Sabine Sarnacki

**Affiliations:** ^1^Department of Surgery, St. Jude Children's Research Hospital, Memphis, TN, United States; ^2^Learning Planet Institute, Université de Paris, Paris, France; ^3^Department of Paediatric Surgery, Hôpital Universitaire Necker-Enfants Malades, Université de Paris, Paris, France

**Keywords:** pediatric tumors, pediatric oncology, pediatric surgical oncology, pediatric cancer, translational surgery

Pediatric surgical oncology (PSO) represents one of the most challenging sub-specialties in the field of pediatric surgery, due to potentially life-threating procedures and the wide field of presentation and location of children's solid tumors.

In this Research Topic of Frontiers in Pediatrics we selected different studies covering both technical and strategical innovations which are necessary for a modern surgical management of pediatric solid tumors.

The first requirement for the optimal treatment of pediatric cancer is a well-trained surgeon in pediatric oncology; Losty in “*Training in Pediatric Surgical Oncology*” highlights the current required skills and related challenges in a worldwide setting of centers with a relative low case-load due to the rarity of pediatric malignancy.

To provide a structured surgical training according to the different tumors' location, vascular, thoracic and urology rotations are encouraged in the 1st years of residency, followed by a specific training in high volume centers with established international fellowship program in PSO (1).

Societies with special commitment in PSO (such as the International Society of Pediatric Surgical Oncology-IPSO) are promoting international fellowship programs and the current trend of improved centralization will help young surgeons to be trained in high-volume centers (2).

Pediatric tumors Show a heterogeneity in their clinical presentation, Persano et al. in “*Burned-Out Testicular Tumors in Adolescents: Clinical Aspects and Outcome*” describe one of these challenging situations. The authors define the clinical presentation of the burned-out testicular tumors as a well-distinct entity, with histology peculiarities in absence of local testicular symptoms, underlying their poor prognosis.

Other than clinical heterogeneity, surgeons should know the potential pitfalls of radiology presentation pf pediatric tumors at diagnosis. Carvalho et al. in “*Diagnostic Errors in Wilms*' *Tumors: Learning From Our Mistakes*” shares the experience and diagnosis complications in one of the largest Pediatric Oncology Institution of America Latina.

Authors emphasize the importance of a multidisciplinary team and the integration of biology in addition with radiologic findings to reach a high diagnostic accuracy for Wilms tumors.

Multidisciplinary strategy has a fundamental role in PSO as described by Theilen et al. in “*Multidisciplinary Treatment Strategies for Wilms Tumor: Recent Advances, Technical Innovations and Future Directions*,” reviewing the role of imaging technology and genetics in clinical practice.

Once the diagnosis is confirmed and neo-adjuvant chemotherapy is initiated, several complications can be observed, some of them requiring surgical evaluation as described in “*Hemorrhage During Induction Chemotherapy in Neuroblastoma: Additional Risk Factors in High-Risk Patients*” by Voglino et al., reporting the effectiveness of conservative treatment for localized hemoperitoneum and thoracic drain placement for hemothorax.

Studies of large cohort of children with specific tumors included in national or international protocols can modify the surgical strategy as described by Machavoine et al. in “*Locoregional Control and Survival in Children, Adolescents, and Young Adults With Localized Head and Neck Alveolar Rhabdomyosarcoma—The French Experience*,” enhancing the role of lymph node surgery and secondary resection of the primary tumor and their positive influence on event-free survival (EFS) for alveolar rhabdomyosarcoma.

Qi and Zhan in “*Roles of Surgery in the Treatment of Patients With High-Risk Neuroblastoma in the Children Oncology Group Study: A Systematic Review and Meta-Analysis*” discuss another important Research Topic about the intraoperative surgical strategy such as resection's completeness. The meta-analysis they provided confirms the recent studies regarding the positive influence of the extent of resection on EFS in High Risk Neuroblastoma (3, 4).

Surgical technique advances allow to change and extend indications as reported by Garnier et al. in “*Case Report: Cytoreductive Surgery and Hyperthermic Intraperitoneal Chemotherapy Application in Intraperitoneally Disseminated Inflammatory Myofibroblastic Tumor and in the Youngest Patient in the World: New Indication and Modification of Technique*.”

Authors describe an extension of the scope of indications of intraperitoneal chemotherapy (usually reserved for children>2 years old and desmoplastic tumors) to a myofibroblastic tumor with a peritoneal carcinomatosis, with excellent oncology outcome at low term follow up after 12 months. A modified hyperthermic intraoperative cytoreductive surgery is reported, with a reduced intraperitoneal normothermic infusion(30 min) and a modified dosage of doxorubicin.

Over the years, minimally invasive surgery (MIS) gained popularity in PSO (5); Ngo et al. in “*Case Report: Transoral Endoscopic Thyroidectomy via Vestibular Approach in Pediatric Thyroid Cancer*” provide an example of the increasing tendency to extend the application of MIS to several types of tumors locations, reporting for the first time an innovative approach in pediatric population providing didactic intraoperative views and trocar positioning description. Authors discuss their novel approach providing a comprehensive technical description and their surgical results.

Vatta et al. in “*Robotics-Assisted Pediatric Oncology Surgery—A Preliminary Single-Center Report and a Systematic Review of Published Studies*” shows the state of the art of a recent evolution of MIS by describing the application of robotics in PSO, with promising results in recently published cohorts of pediatric patients treated for different solid tumors (6).

Pediatric surgery recently benefits from several technology advances as described by Privitera et al. in “*Above and Beyond Robotic Surgery and 3D Modeling in Pediatric Cancer Surgery*.” Authors present a comprehensive review of different techniques for loco-regional intraoperative cancer treatment such as photodynamic therapy and near-infrared photoimmunotherapy, describing their promising results in adult surgical oncology, with a potential application in children.

These innovative local treatments are currently available for adults, but not for children due to the much lower incidence of solid tumors in pediatric population and the subsequent difficulties to design clinical trials based on evidence medicine.

In addition, this review describes the theoretical basis of optical imaging improvement in surgery, including radio, spectroscopy and fluorescent-guided surgery (FGS).

FGS is one of the most promising technology advances in PSO and Abdelhafeez et al. with their original article “*Indocyanine Green–Guided Pediatric Tumor Resection: Approach, Utility, and Challenges*” describe the currently largest cohort of pediatric patients in literature who benefit from FGS. Authors describe the feasibility of FGS and the current limitations of this technique represented by tissue-penetration and background noise of adjacent organs with current fluorophore probes.

Surgeons must be aware of tumor's risk of local relapse after surgery, Pelizzo et al. in “*Proliferation Pattern of Pediatric Tumor-Derived Mesenchymal Stromal Cells and Role in Cancer Dormancy: A Perspective of Study for Surgical Strategy*” study investigate the role of mesenchymal stromal cells as a potential risk factor for local relapse, defining their role in cancer cells dormancy, in addition to their well-known role on drug sensitivity and the subsequent tumor progression and metastasis.

Sundquist et al. provide an example of multidisciplinary scientific collaboration in PSO with the study protocol presentation “*A Phase II Trial of a Personalized, Dose-Intense Administration Schedule of 177Lutetium-DOTATATE in Children With Primary Refractory or Relapsed High-Risk Neuroblastoma–LuDO-N*.” This study protocol involves different subspecialities (nuclear radiologist, oncologist, surgeons, and pathologist) in a common project with the aim of studying an alternative molecular radiotherapy for the most challenging Neuroblastoma presentations (refractory and relapsed high-risk neuroblastoma), in order to improve event free and overall survival rate in high-risk patient population (7).

Survivorship improvement of childhood cancers highlights the functional sequelae impact of chemotherapy and surgery (8).

The results of “*Special Considerations for Tympanoplasty Type I in the Oncological Pediatric Population: A Case-Control Study*” by Richard et al. raise the problem of chronic tympanic membrane perforation in children who survive after cancer treatment, providing surgical optimized strategies in terms of timing and technique.

Young You et al. in “*Considerations for Balance Between Fundamental Treatment and Improvement of Quality of Life of Pediatric Thyroid Cancer Patient: Comparative Analysis With Adult Using Propensity Score Matching*” further study the postoperative quality of life in children with thyroid malignancy, reporting promising outcomes and suggesting to limit total thyroidectomy when is safe and feasible for children, allowing a best functional outcomes.

This Research Topic and all these manuscripts illustrate the current advances on surgical management of pediatric malignancies, highlighting the role of a multidisciplinary approach and the emerging impact and effectiveness of translational surgery, a surgical discipline that serves as bridge across basic science, technology innovations, different medical specialty, and surgery.

## Author contributions

Both authors listed have made a substantial, direct, and intellectual contribution to the work and approved it for publication.

## Conflict of interest

The authors declare that the research was conducted in the absence of any commercial or financial relationships that could be construed as a potential conflict of interest.

## Publisher's note

All claims expressed in this article are solely those of the authors and do not necessarily represent those of their affiliated organizations, or those of the publisher, the editors and the reviewers. Any product that may be evaluated in this article, or claim that may be made by its manufacturer, is not guaranteed or endorsed by the publisher.
